# Beyond the blocking model to fit nanoparticle ZFC/FC magnetisation curves

**DOI:** 10.1038/s41598-018-29501-8

**Published:** 2018-07-24

**Authors:** K. L. Livesey, S. Ruta, N. R. Anderson, D. Baldomir, R. W. Chantrell, D. Serantes

**Affiliations:** 1 UCCS Biofrontiers Center and Department of Physics and Energy Science, University of Colorado – Colorado Springs, Colorado Springs, CO 80918 USA; 2 Department of Physics, The University of York, Heslington, York YO10 5DD United Kingdom; 3 Applied Physics Department and Instituto de Investigacións Tecnolóxicas, Universidade de Santiago de Compostela, E-15782 Campus Vida s/n, Santiago de Compostela, Spain

## Abstract

We consider the probability of a magnetic nanoparticle to flip its magnetisation near the blocking temperature, and use this to develop quasi-analytic expressions for the zero-field-cooled and field-cooled magnetisation, which go beyond the usual critical energy barrier approach to the superparamagnetic transition. The particles in the assembly are assumed to have random alignment of easy axes, and to not interact. We consider all particles to be of the same size and then extend the theory to treat polydisperse systems of particles. In particular, we find that the mode blocking temperature is at a lower temperature than the peak in the zero-field-cooled magnetisation versus temperature curve, in agreement with experiment and previous rate-equation simulations, but in contrast to the assumption many researchers use to analyse experimental data. We show that the quasi-analytic expressions agree with Monte Carlo simulation results but have the advantage of being very quick to use to fit data. We also give an example of fitting experimental data and extracting the anisotropy energy density *K*.

## Introduction

Measurements of the magnetisation of nanoparticles, made in the field-cooled (FC) and zero-field-cooled (ZFC) regimes as a function of temperature, are used to characterise the particle size, size distribution and anisotropy energy^1^. The fact that they are cheap and easy measurements has favoured their broad use, not only to study intrinsic particle properties^2^ but also magnetic ordering^3^ or aggregation conditions^4^, which can be easily identified from the characteristics of the ZFC/FC curves. Furthermore, these measurements are particularly suited to study long-timescale phenomena such as phase segregation^5,6^ or aging and memory effects^7^. Such versatility has led to their use not only for the study of the properties of the magnetic system itself, but also those of the embedding media *via* its effect on the magnetic system^8^. In view of all of the above, it is clear that having a precise and accurate understanding of the features of the ZFC/FC curves is critical for a variety of research areas.

One of the key parameters to characterise particles with uniaxial anisotropy is the “blocking temperature” *T*_*B*_, which is defined as the temperature at which the *average* time for a magnetic nanoparticle moment to escape from an energy well is equal to the characteristic measurement time for the system. Below this temperature, a particle will generally be thermally stable or ‘blocked’ and above this temperature it is classed as superparamagnetic (SPM) since thermal fluctuations are large enough to cause the time-averaged magnetisation to be zero, when there is no external field. The transition from blocked to SPM behaviour is usually taken to occur at a single critical energy barrier. This leads to the assumption of a single blocking temperature *T*_*B*_ at which the transition instantaneously occurs. However, given the stochastic nature of the thermally induced switching, the blocked/SPM transition will have a finite width. Here we aim to modify the blocking formalism to take account of this phenomenon, which is considered to introduce a blocking temperature dispersion for a given nanoparticle size, similar to that considered in ref.^9^. This is an addition to the usual assumption of size polydispersity^10,11^ and should always be included since it is an irreducible contribution to the form of the curve.

In this article, we will present some simple, analytic and quasi-analytic expressions for the FC magnetisation *M*_*fc*_ (*T*) and ZFC magnetisation *M*_*zfc*_ (*T*) for assemblies of magnetic particles with uniaxial anisotropy and random easy axis alignment. The particles are assumed to be non-interacting. The significance of these expressions is two-fold. Firstly, they will allow experimentalists to easily find the mode blocking temperature of particles using a simple fit to their data. Secondly, they elegantly explain some of the key features of these magnetisation curves without the need to use computer simulations, or the requirement that a certain size distribution of particles exists, as has mostly been the case until now.

For the remainder of this introduction, we describe the current methods of fitting ZFC/FC magnetisation curves, so that the reader has the correct background to understand the fitting method presented in the results section.

In the limit of very low temperatures, the magnetisation of a particle is constrained to an energy minimum and the magnetisation of an ensemble of particles can be calculated using methods similar to those of Stoner and Wohlfarth in their famous work on random assemblies^12^. In the limit of high temperatures, the magnetisation in each particle can in theory visit all directions and so a statistical average yields the magnetisation. For a random assembly of particles (the easy axes of the particles point in all directions) the well-known result for the ZFC magnetisation is given by:

1 $$\begin{array}{ccc}\frac{{M}_{{\rm{z}}{\rm{f}}{\rm{c}}}^{{\rm{r}}{\rm{a}}{\rm{n}}{\rm{d}}}(T)}{{M}_{s}} & = & \{\begin{array}{cc}\frac{{\mu }_{0}{M}_{s}H}{3K}, & T < {T}_{B}\\ \coth (\frac{{\mu }_{0}{M}_{s}Hv}{{k}_{B}T})-\frac{{k}_{B}T}{{\mu }_{0}{M}_{s}Hv}, & T > {T}_{B},\end{array}\\  & \equiv  & \{\begin{array}{cc}\frac{{\mu }_{0}{M}_{s}H}{3K}, & T < {T}_{B}\\ {\mathscr{L}}(\frac{{\mu }_{0}{M}_{s}Hv}{{k}_{B}T}), & T > {T}_{B},\end{array}\end{array}$$

where *M*_*s*_ is the saturation magnetisation, *μ*_0_ is the permeability of free space, *H* is the small applied field, *K* is the uniaxial anisotropy energy density, *v* is the volume of a particle, *k*_*B*_ is Boltzmann’s constant, and *T* is temperature. For SPM nanoparticles the magnetisation is given by the Langevin function ${\mathscr{L}}$ and for small values of the argument (linear response regime), ${\mathscr{L}}(x)\sim \frac{x}{3}$. We note that the above expression reflects the critical energy barrier approximation: a single, well-defined blocking temperature represents the transition from blocked to SPM behaviour.

For the corresponding FC magnetisation, we can assume that the magnetisation becomes frozen for temperatures *T* < *T*_*B*_, in other words Eq. (1) becomes

2 $$\frac{{M}_{{\rm{f}}{\rm{c}}}^{{\rm{r}}{\rm{a}}{\rm{n}}{\rm{d}}}(T)}{{M}_{s}}=\{\begin{array}{cc}{\mathscr{L}}(\frac{{\mu }_{0}{M}_{s}Hv}{{k}_{B}{T}_{B}}), & T < {T}_{B}\\ {\mathscr{L}}(\frac{{\mu }_{0}{M}_{s}Hv}{{k}_{B}T}), & T > {T}_{B}\end{array}.$$

**Figure 1:**
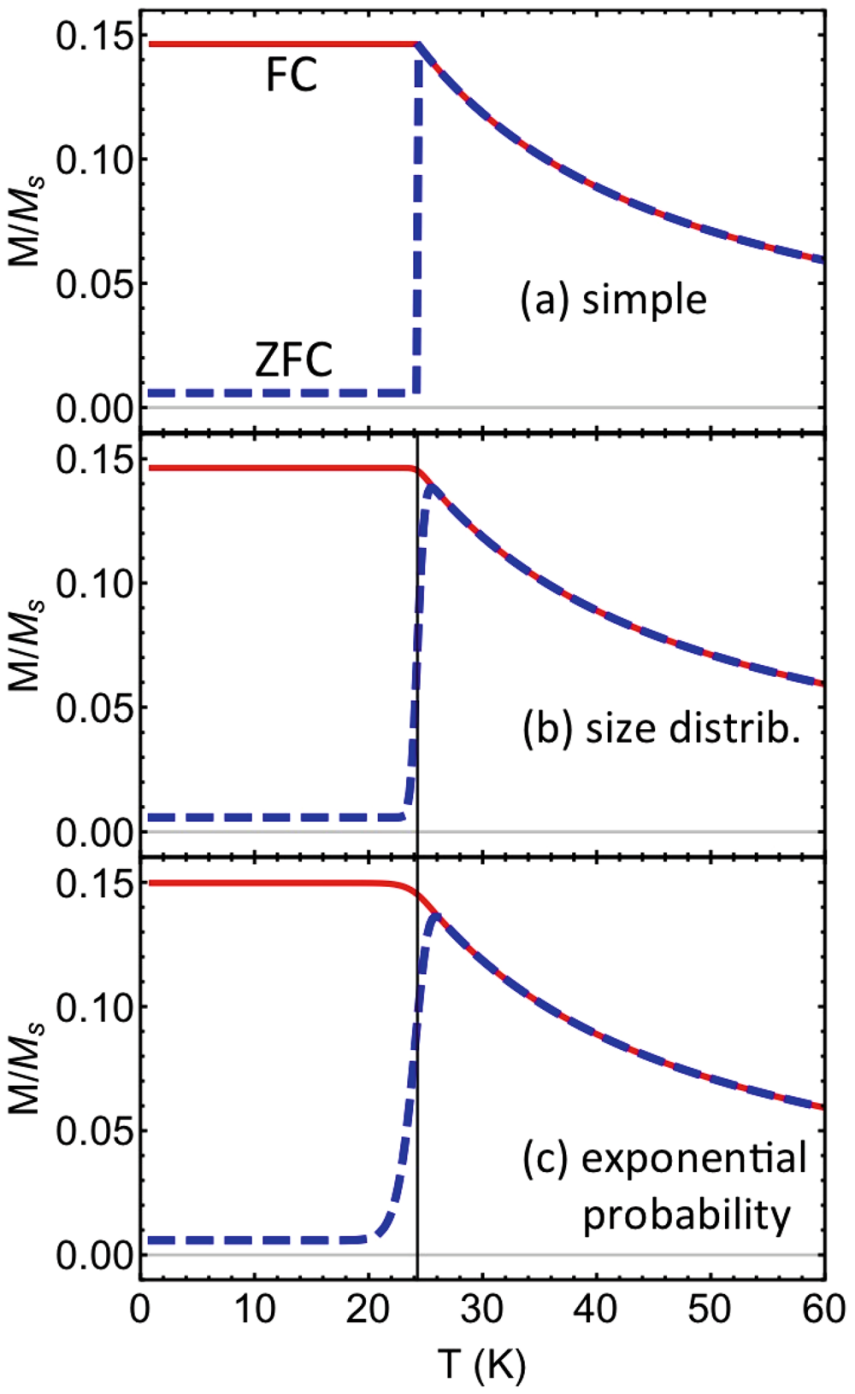
The reduced magnetisation as a function of temperature for a field-cooled (FC, solid lines) and zero-field-cooled (ZFC, dashed lines) random assembly of particles, calculated three different ways. Panel (a) shows the reduced magnetisation calculated using Eqs (1 and 2). Fe_2_O_3_ particles all with 5 nm radius are considered (*M*_*s*_ = 281 kA/m and *K* = 16 kJ/m^3^) with a weak applied field *H* = 10 Oe (796 A/m). These parameters are chosen to match ref.^13^. In panel (b), the magnetisation curves are re-drawn using a log-normal distribution of particle sizes with dispersion *σ* = 0.02 using Eqs (5 and 6) and the sharp peak is smoothed out. In panel (c) our weighted-probability expressions, to be developed later in the text in Eqs (10 and 11), are drawn for particles of a single size and show a similar shape to the plots in panel (b) where a size distribution is assumed. The measurement time is taken to be ln (*τ*_*m*_/*τ*_0_) = 25. The vertical line in panels (b) and (c) indicates the mode blocking temperature, to the left of the ZFC peak.

Plots of these expressions for the ZFC (dashed line) and FC (solid line) magnetisation are shown in Fig. 1(a). Parameters appropriate for 5 nm-radius Fe_2_O_3_ particles were used, following ref.^13^ and are quoted in the figure caption. In Fig. 1(a), one can see discontinuities in the magnetisation and its derivative at the blocking temperature, which are not seen in experiments. Experimental curves are instead smoothly varying, like those shown in Fig. 1(b). These smoother curves in Fig. 1(b) are calculated using a distribution of particle sizes, which we will explain next.

One way that one can use the discontinuous Eqs (1 and 2) to fit data is to take into account that there is a range of particle sizes and therefore a range of blocking temperatures in particle assemblies. The average Néel time for a particle to flip from one well to another is given by

3 $$\tau ={\tau }_{0}\,\exp \,(\frac{Kv}{{k}_{B}T}),$$

where *τ*_0_ is a characteristic attempt time, typically taken as around 10^−9^ s. Note that the application of a magnetic field can change the height of the energy barrier so small fields are assumed here and by most authors. By setting the time *τ* equal to the characteristic time to make a measurement *τ*_*m*_, we define “the” blocking temperature for an experiment according to

4 $${T}_{B}=\frac{Kv}{{k}_{B}\,\mathrm{ln}\,({\tau }_{m}/{\tau }_{0})},$$

where the natural logarithm term is roughly 25 for most quasi-static experiments. One can see that the blocking temperature scales with the particle volume. Typically, particle sizes follow a log-normal distribution with the probability to have a particle with volume between *v* and *v* + *dv* given by *ρ*_*N*_ (*v*)*dv* with

5 $${\rho }_{N}(v)=\frac{1}{v\sigma \sqrt{2\pi }}\exp (\,-\,\frac{{{\rm{l}}{\rm{n}}}^{2}(v/{v}_{0})}{2{\sigma }^{2}}),$$

where *v*_0_ is the peak or mode of the distribution and *σ* (unitless) gives the volume spread. Note that the volume spread is related to the spread in diameters *σ*_*d*_ according to *σ* = 3*σ*_*d*_. Therefore the ZFC magnetisation is given by a weighted sum^10,11,14^

6 $$\frac{{M}_{{\rm{zfc}}}^{\mathrm{log}}(T)}{{M}_{s}}=\frac{{\mu }_{0}{M}_{s}H}{3{k}_{B}T}\frac{1}{{v}_{av}}{\int }_{0}^{{v}_{m}(T)}{v}^{2}{\rho }_{N}(v)dv+\frac{{\mu }_{0}{M}_{s}H}{3K}\frac{1}{{v}_{av}}{\int }_{{v}_{m}(T)}^{\infty }v{\rho }_{N}(v)dv,$$

where *v*_*m*_ (*T*) = (*k*_*B*_*T*)/(*K* ln (*τ*_*m*_/*τ*_0_)) is the critical volume separating superparamagnetic and ferromagnetic particles at a given temperature *T*, and *v*_*av*_ is the average particle volume in the assembly. A similar expression is found for the FC magnetisation^10^. We note that the distribution function *ρ*_*N*_ (*v*) differs from the often used volume fraction distribution *f* (*v*) which represents the magnetic volume contributed by nanoparticles with volumes between *v* and *v* + *dv*. Equivalence of the two approaches is demonstrated by El Hilo and Chantrell^15^ based on the relationship *f* (*v*) ~ *vρ*_*N*_ (*v*). Although derived in^15^ for the lognormal distribution, the result is completely general and any size distribution can be used in place of *ρ*_*N*_. Calculated FC and ZFC curves, using Eq. (6) and the equivalent equation for the FC curve, are plotted in Fig. 1(b) so as to compare to those without particle size distribution in Fig. 1(a). Again, Fe_2_O_3_ particles are considered with a very narrow size distribution given by *σ* = 0.02, around a peak 5 nm radius.

One might naively assume, looking at Fig. 1(a), that the mode blocking temperature for the system occurs where there is a peak in the ZFC magnetisation curve. But in actual fact, one finds in Fig. 1(b) that the mode blocking temperature (corresponding to the mode particle size) is actually just to the left (lower temperatures) than the peak. It is indicated by a vertical line at *T* = 24 K for this system. This is because the magnetisation is so much larger for unblocked particles (high temperatures) than for blocked particles (low temperatures) that the weighted sum given by Eq. (6) is skewed towards high temperatures. Of course, this is only true in the transition region of temperatures, because the magnetisation then decreases at higher temperatures due to thermal averaging.

Recently, different methods of estimating the blocking temperature *T*_*B*_ of particles were compared^13^. Those authors found that the best estimate for the blocking temperature was the point at which the rate of change of the difference (*M*_*zfc*_−*M*_*fc*_) as a function of temperature is maximum, which occurs again to the left of the peak. This was supported by simulations and experimental measurements^13^ and by the work of others^9,16^.

In the Results section we will show that these observations of a smoothed-out ZFC peak and of a (mode) blocking temperature to the left of the peak can both be recovered for assemblies of particles that are all the *same size*, using our analytic expressions. We compare the analytic expressions to results of kinetic Monte Carlo simulations for single-sized magnetic particles in order to support our results. This represents an irreducible thermodynamic contribution to the blocking temperature distribution. We also compare our results for polydisperse samples to experimental ZFC/FC data.

## Results

### Expressions for FC and ZFC magnetisation of monodisperse samples

We begin by returning to the definition of the blocking temperature as the temperature where *on average* a particle’s magnetisation can escape its energy minimum. We will loosely call this a “flip.” If one assumes that flips occur at a constant average rate as defined by the Néel time *τ* in Eq. (3), and each flip is independent (ie. a Poisson process), then the probability of flipping between times *t* and *t* + *dt* is given by an exponential distribution function

7 $${\mathscr{P}}(t)=\frac{1}{\tau }{e}^{-t/\tau }\mathrm{.}$$

In measurements, we are interested in the probability that there will be a flip within a measurement time *τ*_*m*_, and so are interested in the cumulative probability

8 $$p\equiv P({\rm{flip}}\,{\rm{within}}\,{\rm{time}}\,{\tau }_{m})=1-{e}^{-{\tau }_{m}/\tau }.$$

Instead of writing this in terms of waiting times, we can substitute in the Néel time (Eq. (3)) and the definition of the blocking temperature in terms of the measurement time (Eq. (4)) to get a probability in terms of temperature, namely

9 $$\begin{array}{rcl}p(T,\,v) & = & 1-\exp (-\frac{{e}^{Kv/{k}_{B}{T}_{B}}}{{e}^{Kv/{k}_{B}T}})\\  & = & 1-\exp (-{e}^{\frac{Kv}{{k}_{B}}[\frac{1}{{T}_{B}}-\frac{1}{T}]})\mathrm{.}\end{array}$$

In other words, *p* (*T*,*v*) is the probability for a flip within the experimental measurement time at temperature *T*.

Notice that the probability to flip over a barrier depends on the material properties *K* and *v*, plus on the measurement time *τ*_*m*_ through Eqs (3 and 8). When a large magnetic field is applied, this probability to flip would also depend on the applied field strength, on the saturation magnetisation, and on the magnetic damping constant (rate of relaxation), as found by Brown^17,18^. For this reason, we consider only very low fields (*μ*_0_*M*_*s*_*Hv* ≪ *vK*) in this article.

This probability Eq. (9) can be used to weight the expressions for the blocked (low temperature) and unblocked (high temperature) expressions for the ZFC and FC magnetisation given in Eqs (1 and 2) respectively. For the ZFC magnetisation we get

10 $$\begin{array}{ccc}\frac{{M}_{{\rm{z}}{\rm{f}}{\rm{c}}}^{\text{mono}}(T)}{{M}_{s}} & = & (1-p(T,\,v))\frac{{\mu }_{0}{M}_{s}H}{3K}+p(T,\,v)\,{\mathscr{L}}(\frac{{\mu }_{0}{M}_{s}Hv}{{k}_{B}T}).\end{array}$$

For the FC magnetisation the weighted sum is a little more complicated because the value of the “frozen in” magnetisation depends on the fraction of particles that become blocked at various temperatures, depending in turn on the probability to flip given in Eq. (9). In other words, there is a dispersion in the effective blocking temperature and one must integrate over the corresponding probability density to get the correct low-temperature magnetisation, namely

11 $$\frac{{M}_{{\rm{f}}{\rm{c}}}^{\text{mono}}(T)}{{M}_{s}}={\int }_{T}^{{\rm{\infty }}}\xi ({T^{\prime} }_{B},\,v)\,{\mathscr{L}}(\frac{{\mu }_{0}{M}_{s}Hv}{{k}_{B}{T}_{B^{\prime} }})d{T^{\prime} }_{B}+p(T,\,v)\,{\mathscr{L}}(\frac{{\mu }_{0}{M}_{s}Hv}{{k}_{B}T}),$$

where the probability density *ξ*(*T*′, *v*) to be blocked at a temperature *T*′ is defined as

12 $$1-p(T,\,v)\equiv {\int }_{T}^{\infty }\xi (T^{\prime} ,\,v)dT^{\prime} \mathrm{.}$$

Eqs (10) (ZFC, dashed line) and (11) (FC, solid line) are plotted in Fig. 1(c). First, note that including the probability to flip in the analytic model [Fig. 1(c)] smoothes out the discontinuous transition that occurs at the blocking temperature when this probability is ignored [Fig. 1(a)]. In fact, the shapes of the magnetisation curves are very similar to those in the case of using a log-normal distribution of particle sizes to calculate *M* [Fig. 1(b)]. However, it should be noted that the log-normal size distribution used to make this plot is very narrow (*σ* = 0.02) which corresponds roughly to almost all particles having radii between 4.75 and 5.25 nm. Such a narrow distribution was chosen to show the similarity in shape between the two methods of fitting and to show that a distribution of particle sizes is not the only contribution to the smoothed-out ZFC peak.

Secondly, it can be seen in Fig. 1(c) that the blocking temperature is lower than the peak in the ZFC curve, and this occurs without the need to incorporate a distribution of particle sizes. The theory therefore provides a physical reason for why the blocking temperature occurs near the inflection point in the ZFC curve: there is a finite probability for particles to become unblocked and the unblocked particles contribute more to the magnetisation than the blocked ones. Therefore, the blocking temperature corresponding to the mean time to flip is less than the point of maximum ZFC magnetisation.

In fact, we have confirmed both numerically and analytically that by evaluating where *d*^2^(*M*_fc_ − *M*_zfc_)/*dT*^2^ = 0 gives an excellent estimate for *T*_*B*_, in agreement with previous studies^9,13,16^. Analytically, this involves making Taylor expansions for *p* (*T*) and ${\mathscr{L}}$ in Eqs (10 and 11) in the relevant limits. This method of determining the blocking temperature has been described elsewhere. But once again, note that this result falls out of our theory without the need to consider a distribution of particle sizes.

**Figure 2:**
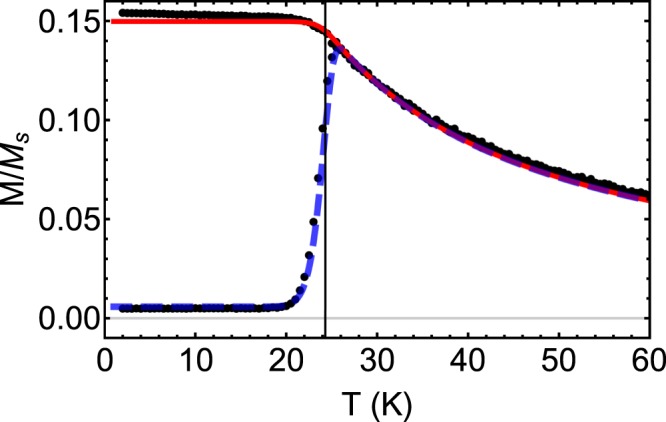
Comparison of kinetic Monte Carlo simulation results (black dots) and results of Eqs (10) (ZFC, blue dashed line) and (11) (FC, red solid line), for a monodisperse assembly of 5 nm radius Fe_2_O_3_ particles. The parameters used are the same as in Fig. 1.

The results of the exponential-probability expressions in Eqs (10 and 11) look very similar to the results produced using rate-equation simulations, also assuming no particle size-distribution^13,19^. To confirm that the simple analytic and semi-analytic expressions match simulation results, we performed kinetic Monte Carlo simulations using the same parameters that were used to make Fig. 1. The results of the kinetic Monte Carlo calculations are shown by black dots in Fig. 2. One can see that the model (red and blue lines) and the numerical kinetic Monte Carlo results match well at all temperatures, supporting the expressions developed here.

### Size distributions

With the expressions established above for a single particle size, it is simple to extend the analytic expression (ZFC) and semi-analytic expression (FC) to include size distributions of particles. Using the log-normal distribution of volumes *ρ*_*N*_ (*v*) in Eq. (5), a weighted sum over volumes is used and replaces Eq. (6), namely

13 $$\begin{array}{c}\frac{{M}_{{\rm{z}}{\rm{f}}{\rm{c}}}(T)}{{M}_{s}}=\frac{1}{{v}_{av}}{\int }_{0}^{{\rm{\infty }}}dv\,v\,{\rho }_{N}(v)\{(1-p(T,\,v))\frac{{\mu }_{0}{M}_{s}H}{3K}+p(T,\,v)\,{\mathscr{L}}(\frac{{\mu }_{0}{M}_{s}Hv}{{k}_{B}T})\},\end{array}$$

and similar for the FC curve we have

14 $$\begin{array}{c}\frac{{M}_{{\rm{f}}{\rm{c}}}(T)}{{M}_{s}}=\frac{1}{{v}_{av}}{\int }_{0}^{{\rm{\infty }}}dv\,v\,{\rho }_{N}(v)\{p(T,\,v)\,{\mathscr{L}}(\frac{{\mu }_{0}{M}_{s}Hv}{{k}_{B}T})+{\int }_{T}^{{\rm{\infty }}}\xi ({T^{\prime} }_{B},\,v)\,{\mathscr{L}}(\frac{{\mu }_{0}{M}_{s}Hv}{{k}_{B}{T}_{B^{\prime} }})d{T^{\prime} }_{B}\},\end{array}$$

Note that *v*_*av*_ is the average volume of the distribution and can be related to the mode/peak volume according to ${v}_{av}={v}_{0}{e}^{{\sigma }^{2}\mathrm{/2}}$.

We pause to consider the difference between Eqs (10,11), and Eqs (13 and 14). The first set of equations we developed (Eqs (10,11)) considered a dispersion of blocking temperatures through the finite flipping probability, with all other properties of the nanoparticles equal in the ensemble (anisotropy, volume, energy barrier). This is a key difference from previous works. The second set of equations (Eqs (13,14)) consider a dispersion of blocking temperatures at each volume, plus a dispersion in the size of particles. This is a more realistic scenario. One could further extend our methods to consider a dispersion in anisotropy constants as well, and even a dispersion of energy barriers due to the relative orientation of the applied field plus dipolar interactions between particles. That is beyond the scope of this work.

The weighted sums over volume in Eqs (13 and 14) can be converted into weighted sums over corresponding blocking temperatures *θ* using Eq. (4). This gives numbers that make the numerical integration and therefore fitting to experimental data quicker and easier to do. By making the correspondence that “the” blocking temperature of a sample *T*_*B*_ corresponds to the mode or peak of the volume distribution, then we have

15 $$\begin{array}{ccc}\frac{{M}_{{\rm{f}}{\rm{c}}}(T)}{{M}_{s}} & = & \frac{1}{{e}^{{\sigma }^{2}/2}}{\int }_{0}^{{\rm{\infty }}}d\theta \,\frac{\theta }{{T}_{B}}f(\theta )\\  &  & \times \,\{p(T,\,\theta )\,{\mathscr{L}}(\frac{25{\mu }_{0}{M}_{s}H\theta }{3KT})+\,{\int }_{T}^{{\rm{\infty }}}\xi ({T^{\prime} }_{B},\,\theta )\,{\mathscr{L}}(\frac{25{\mu }_{0}{M}_{s}H\theta }{K{T}_{B^{\prime} }})d{T^{\prime} }_{B}\},\end{array}$$

where the distribution over blocking temperatures is

16 $$f(\theta )=\frac{1}{\theta \sigma \sqrt{2\pi }}\exp (\,-\,\frac{{{\rm{l}}{\rm{n}}}^{2}(\theta /{T}_{B})}{2{\sigma }^{2}}).$$

Note that the double-integration in Eq. (15) makes this a more difficult expression to fit to experimental data.

**Figure 3:**
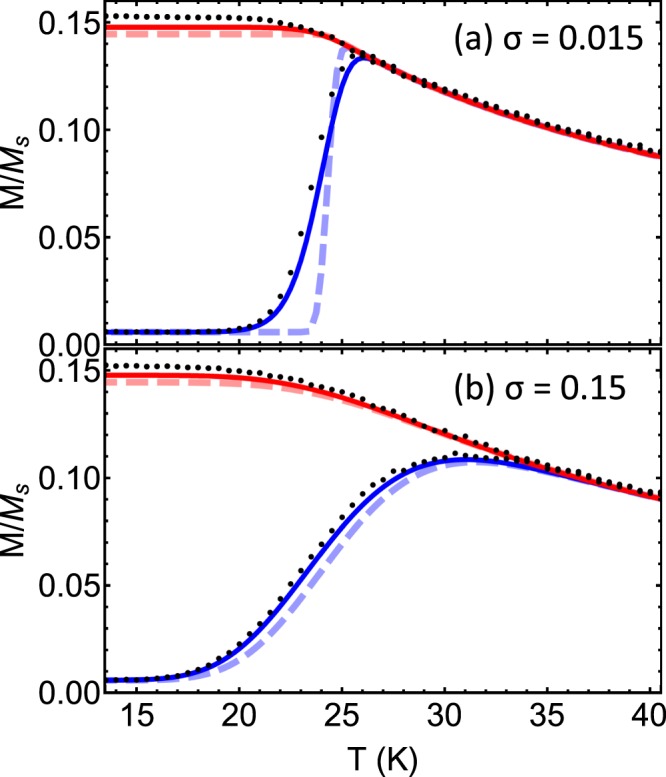
The ZFC (blue) and FC (red) magnetisation curves for magnetite particles with a mode radius of 5 nm and a spread of volumes (**a**) *σ* = 0.015 and (**b**) *σ* = 0.15, in an applied field 10 Oe (796 A/m). The solid curves give the results of using our Eqs (13 and 14). Dashed lines show the results of using Eq. (6) and the corresponding FC equation, given in refs^10,11^. The black dots show kinetic Monte Carlo results.

In Fig. 3, the same nanoparticle parameters are used as in the previous figures to plot the ZFC and FC curves according to Eqs (13) and (14), but a volume distribution of (a) *σ* = 0.015 and (b) *σ* = 0.15 is used. The results of the new fitting process presented in this work are shown by the solid lines and the results of the well-known method to fit, namely Eq. (6) and similar for the FC curve, are shown by the dashed lines. Kinetic Monte Carlo results are shown by the black dots. Notice that in both panels of Fig. 3, the kinetic Monte Carlo results match better with our expressions than with the well-known method.

In panel (a), it is shown that the difference between the two fitting methods is significant for small polydispersity, near the ZFC peak. It is seen that for large polydispersity (panel (b)), the two fitting methods produce almost identical results. In other words, the spread in sizes and therefore blocking temperatures of particles, swamps the spread in the blocking temperature due to particles having finite probability to flip. This implies that the new method is really only crucial to consider for samples with very narrow size distributions. However, the new fitting method presented here gives slightly larger values for the FC magnetisation at low temperatures. This can be explained because there is a larger “frozen in” magnetisation when a dispersion in blocking temperatures is considered. There is also a slight shift to lower temperatures in the blocking region for the ZFC curve for our method, compared to the existing fitting method.

In Fig. 3 the mode blocking temperature calculated from Eq. (4) is *T*_*B*_ = 24 K. Notice in panel (b) that the peak in the ZFC curve is at *T* = 31 K. Using the peak to estimate the mode blocking temperature therefore represents an error of 23%. The ZFC peak moves to higher temperatures for higher polydispersity meaning the error gets worse. Those researchers using the peak to estimate the mode blocking temperature in order to ascertain the anisotropy energy of particles are therefore introducing an unnecessary source of error.

**Figure 4:**
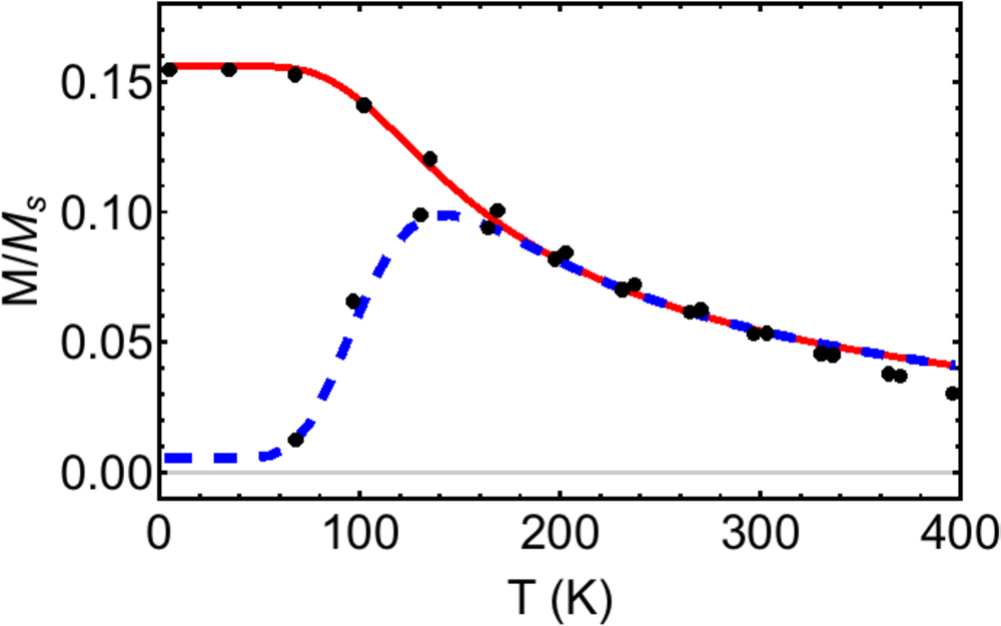
The ZFC-FC experiment data (dots) taken from ref.^1^ for Fe_3_O_4_ particles with mode radius 7 nm, in a field of 10 Oe (796 A/m). Overlaid is our best fit to the data, using Eqs (14) (FC, solid line) and (15) (ZFC, dashed line), with only the anisotropy constant *K* used as a fitting parameter.

To further emphasise this point, we have taken real experimental data from ref.^1^ and plotted it in Fig. 4 (dots) along with a fit using Eqs (14) (FC, solid line) and (15) (ZFC, dashed line). The data is for Fe_3_O_4_ particles with a mode radius of 7 nm, a volume spread given by *σ* = 0.246, saturation mass magnetisation given by *M*_*s*_ = 83 A⋅m^2^/kg (corresponding to volume magnetisation 430 kA/m), and the measurement timescale gives ln (*τ*_*m*_/*τ*_0_) = 27. The particles are in a 10 Oe (796 A/m) applied field. These parameters are all taken from ref.^1^ and are extracted from magnetisation loop and dynamic light scattering experiments. The *only* parameter, therefore, used to fit the ZFC/FC data is the anisotropy constant *K*.

In Fig. 4, our best fit to the data is shown, which used a value *K* = 23 kJ/m^3^ input into Eqs (14 and 15). Note that the fit is very good, even though only one fitting parameter is used. This value for the anisotropy energy density equates (using Eq. (4)) to a mode blocking temperature *T*_*B*_ = 96 K. This is consistent with the fact that the mode blocking temperature occurs where *d*^2^(*M*_fc_ − *M*_zfc_)/*dT*^2^ = 0, to the left of the ZFC peak position. Once again, using the position of the ZFC peak to estimate the mode blocking temperature (141 K) leads to an error of 46% in both the blocking temperature and the anisotropy constant. Ref.^1^ estimates *K* = 35.5 kJ/m^3^ if interactions between nanoparticles are ignored, compared to our value of *K* = 23 kJ/m^3^. This is closer to the bulk value of 13.5 kJ/m^3^. The role of interactions between particles is discussed below.

## Discussion

An alternate method to fit ZFC and FC data of randomly oriented nanoparticles, in weak applied fields, is presented that considers the finite probability for a particle to flip over an energy barrier–at all temperatures–within a given measurement time. This equates to considering a dispersion of blocking temperatures, for a constant particle volume, in contrast to existing methods that consider a particle’s volume to be indicative of a specific blocking temperature.

The method was compared to kinetic Monte Carlo simulations and shown to be robust for monodisperse and polydisperse systems. If there is a large spread in particle sizes, the method does not deviate significantly from the current method most researchers use to fit ZFC and FC curves. But when there is a small size distribution, the difference is significant. Moreover, the new method gives an obvious reason for why the mode blocking temperature occurs at the temperature where *d*^2^(*M*_fc_ − *M*_zfc_)/*dT*^2^ = 0, to the left of the ZFC peak position. Using the incorrect assumption that the mode blocking temperature occurs at the peak of the ZFC curve leads to errors in the range of 20–50% in this and any consequent calculation for the particles’ mode size or anisotropy constant *K*. We demonstrate this by fitting to one example of experimental data, with our estimate of *K* differing by 46%.

The semi-analytic expressions developed here resolve a discrepancy between the magnetisation predicted for monodisperse systems of particles using (i) the usual method of fitting (abrupt change in magnetisation at “the” blocking temperature), and (ii) numerical simulations (gradual change in magnetisation with temperature). It may be argued that for large polydispersity, one only needs to keep in mind that the mode blocking temperature occurs where *d*^2^(*M*_fc_−*M*_zfc_)/*dT*^2^ = 0 and then one can continue using the usual fitting methods, rather than the more complicated expressions presented here. However, our method describes accurately an irreducible contribution to the ZFC and FC curves from a dispersion of blocking temperatures, and provides a basis to extend predictions to systems with dispersity in other parameters, such as anisotropy energy density, shape, and local dipolar field strength. Our method may also be extended to systems with some orientational order in the easy anisotropy axes^20^.

Our model does not take into account the role of dipolar interactions between the particles, which is known to add an additional *effective* contribution to the uniaxial anisotropy, for assemblies with randomly oriented easy axes^21,22^. Here we give a crude estimate of the interactions’ effect on the magnetocrystalline anisotropy constant that is extracted from ZFC-FC experiments. The Vogel-Fulcher model^23^ can be used to estimate the intrinsic magnetocrystalline anisotropy *K.* It effectively subtracts the effect of the interactions on the barrier height and lowers the estimate in ref.^1^ from *K* = 35.5 kJ/m^3^ to *K* = 21.25 kJ/m^3^, for the 7 nm-radius particle system discussed in Fig. 4. In that work, the authors used low field *M-H* measurements and the Curie-Weiss law for paramagnetism to estimate a magnetic interaction temperature *T*_0_ = 43.2 K. Then the magnetocrystalline anisotropy can be estimated according to

17 $$K=\frac{{k}_{B}({T}_{B}-{T}_{0})}{v}\,\mathrm{ln}\,({\tau }_{m}/{\tau }_{0}),$$

where *T*_*B*_ is the mode blocking temperature. Using the mode blocking temperature from our fit *T*_*B*_ = 96 K, rather than using the temperature at which the ZFC has its peak, gives *K* = 13.6 kJ/m^3^ when interactions are included according to Eq. (17). This is a reduction from 23 kJ/m^3^ that we quoted before, when the interaction temperature was ignored. Remarkably, this value is almost exactly the bulk value measured for this material, namely *K*_*bulk*_ = 13.5 kJ/m^3^ ^1^. We note that it is not unusual to have nanoparticle magnetocrystalline anisotropy differ from that of bulk, due to surface effects. But the difference may not be as dramatic as some authors suggest, when the mode blocking temperature is estimated correctly.

The rate at which temperature is changed in FC/ZFC experiments can alter the effective mode blocking temperature^24^. Note that Eq. (3) relates the measurement time to the mode blocking temperature, but does not depend on the rate at which the temperature is changed between taking data points. This is beyond the scope of the current work, where we show that even monodisperse particle systems have a mode blocking temperature below the ZFC peak.

The saturation magnetisation and anisotropy constant may vary over the range of temperatures examined, which is not captured in our theory, and will change the shape of the predicted ZFC/FC curves^2^. This may be the reason that there is a discrepancy at high temperatures in Fig. 4 between the theory and experiment (the experimental points lying below the theoretical Curie-like decay curve). This is something to consider in future work.

## Methods

### Kinetic Monte Carlo

The nanoparticle system is modeled using a kinetic Monte-Carlo (MC) approach, which takes into account the behaviour of both the thermally stable and superparamagnetic particles, and is described in detail in ref.^25^. We define a thermally stable particle as one that satisfies *Kv *> *k*_*B*_*T* ln (*τ*_*m*_/*τ*_0_). The equilibrium position of the moment of such a particle in the local field *H*_*loc*_ is calculated using the Stoner-Wohlfarth (SW) model^12^. If the SW model gives two equilibrium positions in the energy landscape, the moment can then jump between these positions with a reversal probability,

18 $${p}_{rev}=1-\exp (\,-\,{\tau }_{m}/\tau ),$$

where *τ* = *τ*_0_*exp* (Δ*E*/(*k*_*B*_*T*)) is the mean time to reverse over an energy barrier Δ*E* (*ψ*, ***H***_***loc***_), where *ψ* is the orientation of the easy-axis relative to the local field. The barrier is calculated using a numerical approximation due to Pfeiffer^26^ where,

19 $${\rm{\Delta }}E(\psi ,\,{{\boldsymbol{H}}}_{{\boldsymbol{loc}}})=Kv{\mathrm{[1}-|{{\boldsymbol{H}}}_{{\boldsymbol{loc}}}|/g(\psi )]}^{\kappa (\psi )},$$

with $g(\psi )={[{cos}^{\mathrm{2/3}}\psi +{sin}^{\mathrm{2/3}}\psi ]}^{-\mathrm{3/2}}$, and *κ*(*ψ*) = 0.86 + 1.14 *g* (*ψ*). Note that in deriving the semi-analytic expressions, the energy barrier did not depend on the local applied field. There is therefore a different assumption here, but with little difference in the results as long as the applied field is small.

SPM behaviour occurs up to large energy barriers, which for a measurement time of 100 s can typically be up to 25*k*_*B*_*T*. Persistence in the SPM behaviour creates difficulties for standard MC approaches due to the unreasonably large number of MC steps that are necessary to achieve equilibrium. By considering the SPM particles with large energy barriers (namely *Kv* > 3*k*_*B*_*T*) as a two-state system, an improved computational approach can therefore be derived^25^. This approach leads to the condition that, if the reversal transition is allowed, the moment is then assigned to either energy minimum with a probability,

20 $${p}_{i}={e}^{-{E}_{i}}/({e}^{-{E}_{1}}+{e}^{-{E}_{2}}),$$

with *i* = 1,2 labeling the minima, thereby ensuring that the population of the two states obeys the Boltzmann distribution in thermal equilibrium^25^. For smaller energy barriers (*Kv* < 3*k*_*B*_*T*), a standard Metropolis algorithm is used, with the angles of the magnetic moment *θ* and *ϕ* being modified randomly^27^. In this case, the energy difference Δ*E* between the new energy state and the previous one is calculated and the moment is then allowed to remain in its new position with the probability $p=\,{\rm{\min }}(\mathrm{1,}\,{e}^{-{\rm{\Delta }}E/{k}_{B}T})$. For thermally stable particles (after determining the relevant minimum), we use standard MC moves to model the thermal equilibrium distribution about the energy minimum.

To compare to the semi-analytic model predictions, we calculate kMC data for *M* versus *T* in a particular way. Each data point–at various temperatures–is calculated by preparing the system of particles at 2 K and using this as the initial configuration to iterate from. This is the data presented in Fig. 3. If we do a simulation where the system has its magnetisation calculated at each temperature as it is heated from 2 K, we find that the magnetisation is unblocked at slightly lower temperatures, which depends on the effective rate of temperature change^24^.

### Data Availability

The datasets generated during and/or analysed during the current study are available from the corresponding author on reasonable request.
